# Concentrations of Formic Acid, Acetic Acid, and Ammonia in Newly Constructed Houses

**DOI:** 10.3390/ijerph17061940

**Published:** 2020-03-16

**Authors:** Norimichi Suzuki, Hiroko Nakaoka, Akifumi Eguchi, Masamichi Hanazato, Yoshitake Nakayama, Kayo Tsumura, Kohki Takaguchi, Kazunari Takaya, Emiko Todaka, Chisato Mori

**Affiliations:** 1 Center for Preventive Medical Sciences, Chiba University, 6-2-1 Kashiwanoha, Kashiwa, Chiba 277-0882, Japan; hnakaoka@faculty.chiba-u.jp (H.N.); a_eguchi@chiba-u.jp (A.E.); hanazato@chiba-u.jp (M.H.); seiken@chiba-u.jp (Y.N.); tsumu-kayo@chiba-u.jp (K.T.); k.takaguchi@chiba-u.jp (K.T.); todakae@faculty.chiba-u.jp (E.T.); cmori@faculty.chiba-u.jp (C.M.); 2 National Institute of Occupational Safety and Health, 6-21-1 Nagao, Tama-ku, Kawasaki 214-8585, Japan; takaya-k@h.jniosh.johas.go.jp; 3 Department of Bioenvironmental Medicine, Graduate School of Medicine, Chiba University, Chuo-ku Inohana 1-8-1, Chiba 263-8522, Japan

**Keywords:** indoor air quality, formic acid, acetic acid, ammonia, sum of volatile organic compounds, newly built houses

## Abstract

Herein, the concentrations of formic acid, acetic acid, and ammonia in samples of indoor air for 47 new houses were measured two weeks after completion. The houses were fabricated with light-gauge steel structures. The measurements were performed in living rooms and bedrooms without furniture and outdoors. Air samples were analyzed using ion chromatography. The mean values were 28 (living room), 30 (bedroom), and 20 μg m^−3^ (outdoor air) for formic acid; 166 (living room), 151 (bedroom), and 51 μg m^−3^ (outdoor air) for acetic acid; and 73 (living room), 76 (bedroom), and 21 μg m^−3^ (outdoor air) for ammonia. The total values of the three substances accounted for 39.4–40.7% of the sum of chemical compound values. The analyzed compounds were indicated by two principal components (PC), PC1 (30.1%) and PC2 (9%), with 39.1% total variance. Formic acid, acetic acid, and ammonia were positively aligned with PC1 and negatively aligned with PC2. Factors such as room temperature, aldehydes, and phthalates were positively aligned with PC1 and negatively aligned with PC2. Furthermore, concentrations of formic acid, acetic acid, and ammonia were significantly and positively correlated with room temperature (*p* < 0.05).

## 1. Introduction

Because of the recent advancement of new, largely insulated, airtight housing that saves energy, there is an increasing concern regarding indoor safety as there is a higher possibility of chemical substances accumulating in these dwellings compared to conventional houses [1,2]. If there is insufficient ventilation, chemical substances may remain indoors for a prolonged period, which can increase the risk of the inhabitants being exposed to chemicals [3,4]. Volatile organic compounds (VOCs) released from building materials, furniture, and personal care products can affect human health. An example of this is building-related symptoms, which can present issues such as sensory irritation, a thickening of mucous membranes, and irregular respiratory symptoms [5,6,7]. The Ministry of Health, Labor and Welfare (MHLW) of Japan has established guideline values for 13 chemical substances and a provisional target value that serves as a limitation for the amount of total volatile organic compounds (TVOC) [8] to prevent detrimental health effects on occupants induced by chemicals in indoor air. For indoor air in newly built houses, however, there are several unregulated VOCs that do not have guideline values [9,10]. There are also reports indicating that these unregulated chemical substances can have adverse health effects [10,11]. Among these unregulated substances, organic acids are observed to be abundant in new houses and with elevated concentrations [12,13]. High levels of formic acid and acetic acid are estimated to have adverse health effects on the public, such as the degeneration of olfactory epithelium (formic acid) and irritation of the upper respiratory tract (acetic acid) [13,14,15]. Ammonia is also considered to cause irritative effects in humans at high concentrations [16]. In the Lowest Concentration of Interest list developed by the European Union (EU-LCI) [17], there is an emission limit value for acetic acid. For ammonia, the Finish Society of Indoor Air Quality and Climate (FiSIAQ) has set guideline values for three categories in office buildings [18]. When the public is exposed to these airborne pollutants, formic acid, acetic acid, and ammonia can cause adverse health problems in humans [19,20,21]. However, in Japan, there are no guideline values for these substances in indoor air, and there are only a few reports of their measurements in newly built houses. In addition, it is difficult to measure and identify these chemical substances accurately, due to the differences in measurement methods for other VOCs and carbonyls.

This study aimed to accurately measure the concentration levels of formic acid, acetic acid, and ammonia, since exposure to them may have an effect on human health.

## 2. Materials and Methods

### 2.1. Study Houses

Overall, 47 houses were selected from 49 houses that were used as test sites for our previous study [22]. Air samples from these locations were captured and analyzed for the presence of organic acids and ammonia. All the houses were newly built by the steel structure method from 2014 to 2016 in Chiba Prefecture, Japan. The approximate dimensions of houses were 138 m^2^ and they had 2.4 bedrooms on average. The mean size of the living room and bedroom areas were 30.44 and 12.34 m^2^, respectively. The floors were covered with laminated flooring, which is a multi-layer synthetic flooring product fused together with a lamination process. The walls were decorated with wallpaper using starch adhesive or painted using water-based paints. Air samples were collected from living rooms, bedrooms, and outside of the houses two weeks after their completion and before occupants moved in, so there were no furniture, detergents, or personal products in the houses. Because these houses were built at different time, measurements of temperature and humidity were simultaneously and continuously recorded during the sampling for 30 min to investigate the relationship between them. 

### 2.2. Sampling and Analysis of Target Compounds in Indoor and Outdoor Air

Air samples were obtained using an active sampling method for 120 min. Before indoor air sampling, rooms were ventilated by opening windows and doors for at least 30 min. Then, all doors and windows were closed for more than five hours. In addition to organic acids and ammonia, VOCs, carbonyls, and semi-volatile organic compounds (SVOCs) were simultaneously collected.

Formic acid, acetic acid, and ammonia were sampled by two connected impingers containing 10 mL of ultrapure water as a sampling liquid. These compounds were identified and quantified using an ion chromatography system Dionex ICS-1600 (Thermo Fisher Scientific Inc., Tokyo, Japan) with isocratic elution. The mobile phase was established at a 1-mL min^−1^ flow rate and applied with 4.5 mM Na_2_CO_3_/0.8 mM of sodium hydrogen carbonate for the analysis of formic acid and acetic acid, and 6 mM of methanesulfonic acid aqueous solution for the analysis of ammonia. Then, 25 μL of sample solution was injected into an analytic column and IonPac AS23 (250 × 4 mm i.d.) that was serially connected with a guard column IonPac AG23 (50 × 4 mm i.d.) and reagent-free ion chromatography (RFIC) suppressor AERS 500 (4 mm) for formic acid and acetic acid. For the analysis of ammonia, IonPac CS17 (250 × 4 mm i.d.), IonPac CG17 (50 × 4 mm i.d.) and CERS 500 (4 mm) were applied as an analytical column, a guard column, and an RFIC suppressor, respectively. The temperature of the oven was set to 30 °C.

For the sampling and measurements of VOCs, carbonyl compounds, and SVOCs, we used Tenax-TA^®^ (Sigma-Aldrich, St. Louis, Missouri, USA), DNPH tube gas (designed for aldehydes and ketones; Shibata Scientific Technology Ltd., Saitama, Japan), and 47-mm Empore C-18FF Disks (3MJapanLtd. TwoHarbors, Minnesota, USA) as samplers, respectively. VOCs were extracted via thermal desorption and analyzed via gas chromatography-mass spectrometry (GC/MS). For the carbonyl compounds analytes, solvent extraction and high-performance liquid chromatography (HPLC) were used. SVOCs were analyzed via GC/MS spectrometry after ultrasonic extraction. The levels of those substances in indoor and outdoor air were previously reported [22]. These measurements were performed in compliance with the “Indoor Air-Sampling strategy for volatile organic compounds (VOCs)” by JIS A 1965 and 1966 [23]. They are based on ISO 16000-5 [24], which was issued as the first edition in 2007, and modified with technical content to reflect the actual situation in Japan.

The measurements of the ventilation rates were performed using the tracer gas method, known as the decay rate method. CO_2_ was generated using dry ice, and the gradual decrease in the concentration from the maximum value over 60 min was measured at 1-min intervals using a CO_2_ concentration meter (TES-1370, Satoshoji Digital, Kanagawa, Japan). We calculated the ventilation rate V according to the formula described in “Standard Methods of Analysis of Sanitary Chemists” [25].

V = 2.303 × VR/t × log {(C1 − C0) / (Ct − C0)}(1)

The above abbreviations are VR: room volume (m^3^), t: the duration of the mechanical ventilation’s operation, Ct: the tracer gas concentration (μg m^−3^ or mL m^−3^) at time t, C1: the initial tracer gas concentration (μg m^−3^ or mL m^−3^), and C0: the outdoor tracer gas concentration (μg m^−3^ or mL m^−3^).

### 2.3. Statics Analysis

Spearman’s rank correlation coefficient and principal component analysis (PCA) were performed using R 3.6.0 [26]. Prior to PCA analysis, all values were standardized using the following equation

z = x − μ/σ,(2)

where μ was the mean and σ was the standard deviation of the variables. PCA was obtained using the R package FactoMineR [27]. The compounds with a limit of quantity (LOQ) of >50% were excluded from the data analysis.

## 3. Results

The mean, standard deviation (SD), maximum, and minimum values of temperature, and the relative humidity of each room investigated in this study, are listed in Table 1. Since air samplings were conducted before the residents moved in, the air conditioner was off during the sampling so that the temperature indoors could reach 7 or 8 °C.

Table 2 demonstrates the levels of formic acid, acetic acid, and ammonia in air samples from living rooms, bedrooms, and outdoor areas in this study and previous studies [22]. These compounds were detected in all the samples analyzed in this study. Mean concentrations of formic acid, acetic acid, and ammonia in the air samples were 28, 166, and 73 μg m^−3^ for living rooms, 30, 151, and 76 μg m^−3^ for bedrooms, and 20, 51, and 21 μg m^−3^ for outside the houses, respectively.

**Figure 1:**
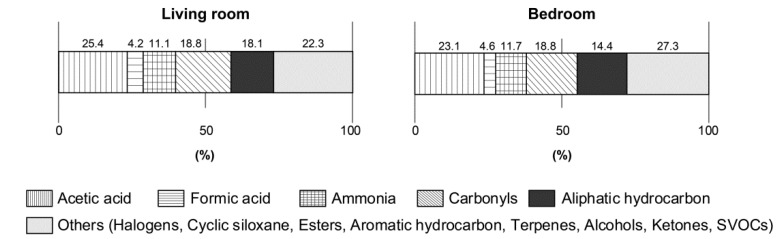
The ratio of acetic acid and formic acid in the sum of volatile organic compounds (VOCs).

The proportion of formic acid, acetic acid, and ammonia in the sum of the VOCs are shown in Figure 1. The sum of the VOCs was the sum of concentrations of formic acid, acetic acid, ammonia, 55 VOCs, 14 carbonyls, and 22 SVOCs. The VOCs were categorized as esters, halogens, alcohols, aromatic hydrocarbons, ketones, terpenes, and cyclic siloxane. In living rooms, formic acid, acetic acid, and ammonia accounted for 4.2%, 25.4%, and 11.1% of the sum of VOCs, respectively. In the bedrooms, the ratios of formic acid, acetic acid, and ammonia in the sum of VOC levels were 4.6%, 23.1%, and 11.7%, respectively.

**Figure 2:**
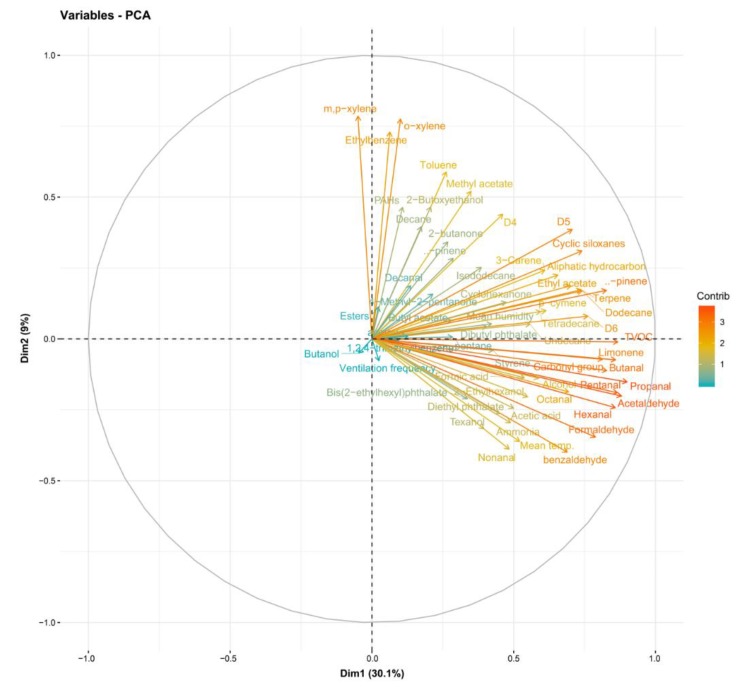
Loading of principal components.

**Figure 3:**
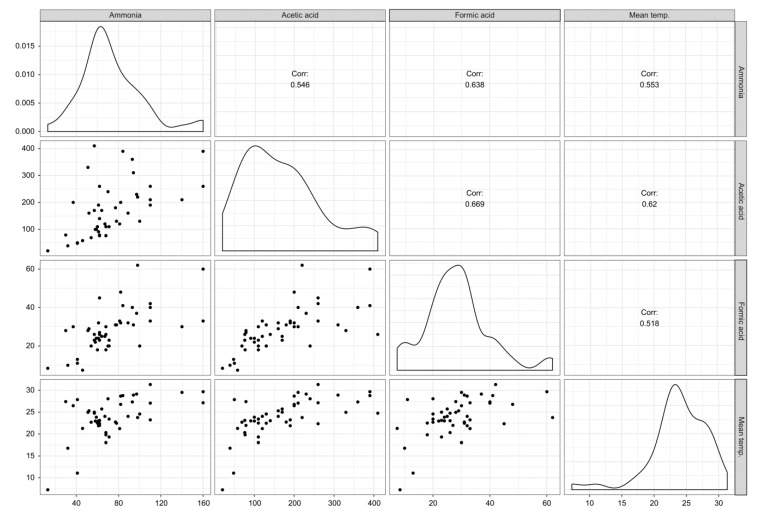
Correlation among acetic acid, formic acid, ammonia, and room temperature. The correlation coefficients are expressed by “*r*”, indicating that there are correlations among them.

The analyzed compounds were indicated by two principal components (PC), PC1 (30.1%) and PC2 (9%), with a total variance of 39.1%. Formic acid, acetic acid, and ammonia were positively aligned with PC1 and negatively aligned with PC2. Room temperature, aldehydes, and phthalates were positively aligned with PC1 and negatively aligned with PC2 (Figure 2). Formic acid, acetic acid, and ammonia were positively correlated with room temperature to a significant degree (*p* < 0.05) (Figure 3).

## 4. Discussion

In this study, we examined the concentrations of formic acid, acetic acid, and ammonia in samples of indoor air from 47 newly built houses with light steel structures two weeks after their completion in the Chiba Prefecture, Japan. In Japan, an indoor air chemical concentration survey was conducted nationwide for 602 randomly selected houses in 2012, 2013, and 2014 [14]. Comparing the results of the two studies, the three substances, formic acid, acetic acid and ammonia, had almost the same concentration levels when averaged across the dwellings. Comparing the results of these two studies is challenging because of differences in area, housing structure, and age, but it may suggest that airborne concentrations of formic acid, acetic acid, and ammonia do not decrease over time. Those substances do not change, possibly because of the increase in occupants’ use or carry-in, or the occurrence of secondary generation. There are few reports on the health effects of these chemical substances on humans, but they have pungent odors. Pungent odors can cause discomfort or mucous membrane irritation [28,29,30]. These substances have been found in abundance in indoor air because the houses were newly built, rendering it necessary to evaluate their health effects on humans.

### 4.1. Formic Acid

Formic acid is a colorless, fuming liquid with a pungent odor at room temperature. Its odor threshold is reported as 0.52–340 ppm (1–640 mg m^−3^) by the American Industrial Hygiene Association [31] and its boiling point is 100.8 °C, and it is very corrosive to the eyes, skin, and respiratory tract [32]. Herein, the indoor/outdoor (I/O) ratio of formic acid was 1.5, indicating that formic acid could be emitted indoors. One mechanism of formic acid generation is thought to be the oxidation process of formaldehyde [33,34]. Because the floors were covered with laminated flooring in the test sites, and the mean value of 15 μg m^−3^ of formaldehyde was detected [22], it is likely that the formic acid in indoor air samples herein was emitted directly from building materials or generated by the reaction of formaldehyde inside or on the building material surface. Its concentration levels in indoor air samples were low (Table 2), and it accounted for a small percentage of the sum of VOCs, from 4.2% to 4.6% (Figure 1), herein. 

### 4.2. Acetic Acid

Acetic acid is a colorless, flammable liquid with a pungent odor. Its odor threshold varies greatly because of the differences in methodologies. Among them, there are reports that it is from 0.006 (14.7 μg m^−3^) [35] to 1.00 ppm (2460 μg m^−3^) [36]. It has been suggested that vapors of acetic acid could have a mild irritative effect at 10 ppm (25 mg m^−3^) for healthy people [15], which is much higher than this study’s result. In this study, the mean values of acetic acid were 166 μg m^−3^ in the living rooms, 151 μg m^−3^ in the bedrooms, and 49 μg m^−3^ outdoors (Table 2). The I/O ratios of acetic acid were 3.4 in the living rooms and 3.1 in the bedrooms, and it is likely that the emission sources were indoor building materials. The mean values of acetic acid accounted for the largest percentage of the sum of VOCs, from 23.1% to 25.4% (Figure 1). The guideline value of acetic acid is set to 1200 μg m^−3^ in the EU-LCI values [17]. LCI values are used to evaluate the emissions from building products into indoor air. In the Japanese Building Standard Law, the guideline value of formaldehyde indoors was derived as per the restrictions of using building materials. In the case where we tentatively used the LCI values with some care in this study, the values detected were lower than those that could affect health.

### 4.3. Ammonia

Ammonia is an inorganic, colorless compound with a pungent odor. Nagata et al. reported its odor threshold as 1.5 ppm (1045 μg m^−3^) [35], and it is designated as a specific malodorous substance under the Offensive Odor Control Law by the Japanese Ministry of the Environment [37]. Ammonia in indoor air is mainly related to human activity, building materials, paint, and personal products. The I/O ratio of its concentration levels in this study were 3.6 in living rooms and 3.5 in bedrooms and higher in indoor air than in outdoor air. Because the air samples were collected indoors without furniture and occupants, the source of the ammonia detected in this study was likely indoor building materials. There are no guideline values for ammonia in Japan, but the Finish Society of Indoor Air Quality and Climate has set guideline values for three categories in office buildings, 30–40 μg m^−3^ [17], and some samples in this study exceeded these guideline values. Further investigation is required to evaluate ammonia in these new types of housing, particularly the adverse effects that ammonia may have on humans.

In this study, the total amount of formic acid, acetic acid, and ammonia accounted for 39.4–40.7% of the sum of VOCs and it was the highest proportion out of them. Some studies report that these odorous substances can cause sensory irritation [38,39,40]. Further investigation into the relationship between these compounds and adverse sensory irritation in humans induced by odor-driven causes is required.

Additionally, this study found that there was a correlation among formic acid, acetic acid, ammonia, and room temperature (Figure 3). These three substances are also related to aldehydes and phthalates (Figure 2). The relationship among these substances most likely indicates shared sources. Considering that water-based polyvinyl acetate (PVA) adhesive emits acetic acid [41], acetic acid detected in the newly built houses herein were likely emitted mostly from construction materials with PVA adhesive. Phthalates are primarily used as plasticizers, and they have become ubiquitous in the developed world [42]. In this study, when sampling chemical substances in indoor air, there were no furniture, appliances, or daily products, so the emission source of phthalates was also estimated to be from the building materials. However, it is unclear and further investigation on the source is desired.

## 5. Conclusions

This study found that the sum of formic acid, acetic acid, and ammonia in indoor air samples from newly constructed houses was the highest out of the sum of VOCs. Further, these substances were correlated with temperature and had some relationship with aldehydes and phthalates. The findings of this study can help to evaluate their exposure to the occupants.

## Figures and Tables

**Table 1:** Indoor environments.

		Living Room				Bedroom				Outdoor			
		Mean	SD (±)	Max	Min	Mean	SD (±)	Max	Min	Mean	SD (±)	Max	Min
Temperature	°C	23.8	5.7	31.3	7.3	25.1	4.3	33.2	8.0	26.5	7.0	37.7	4.4
Relative humidity	%	62.3	14.1	82.2	24.6	58.0	10.5	77.5	24.4	54.3	17.4	94.2	23.4
Ventilation rates	Per hour	1.2	0.5	2.1	0.6	―	―	―	―	―	―	―	―

**Table 2:** Concentrations and frequencies of acetic acid and formic acid compounds.

								Our study (2015–2016)												S. Uchiyama et al. (2015)
		Living Room, n = 47						Bedroom, n = 47						Outdoor, n = 47						n = 602
	LOQ ^(a)^	Mean	SD (±)	Median	Max	Min	Frequency	Mean	SD (±)	Median	Max	Min	Frequency	Mean	SD (±)	Median	Max	Min	Frequency	Median (Summer)
		(μg m^−3^)	(μg m^−3^)	(μg m^−3^)	(μg m^−3^)	(μg m^−3^)	(%)	(μg m^−3^)	(μg m^−^^3^)	(μg m^−^^3^)	(μg m^−^^3^)	(μg m^−3^)	(%)	(μg m^−3^)	(μg m^−3^)	(μg m^−3^)	(μg m^−3^)	(μg m^−3^)	(%)	(μg m^−3^)
Acetic acid	3.0	169	99	160	410	20	100	148	89	130	400	0.8	100	49	37	39	160	ND^(b)^	100	130
Formic acid	3.0	28	11	28	62	7.0	100	30	15	28	91	1.6	100	20	7.1	18	38	5.6	100	28
Ammonia	3.0	73	30	68	160	13	100	77	32	75	160	10	100	21	13	17	59	5.9	100	37

^(a)^ LOQ: limit of quantitation; ^(b)^ ND: Not detected.
